# The association between patient activation and self‐care practices: A cross‐sectional study of an Australian population with comorbid diabetes and chronic kidney disease

**DOI:** 10.1111/hex.12577

**Published:** 2017-07-04

**Authors:** Edward Zimbudzi, Clement Lo, Sanjeeva Ranasinha, Peter G Kerr, Kevan R Polkinghorne, Helena Teede, Timothy Usherwood, Rowan G Walker, Greg Johnson, Greg Fulcher, Sophia Zoungas

**Affiliations:** ^1^ Department of Nephrology Monash Health Clayton Vic Australia; ^2^ Monash Centre for Health Research and Implementation School of Public Health and Preventive Medicine Monash University Clayton Vic Australia; ^3^ Diabetes and Vascular Medicine Unit Monash Health Clayton Vic Australia; ^4^ Department of General Practice Sydney Medical School Westmead University of Sydney Sydney NSW Australia; ^5^ The George Institute for Global Health Camperdown NSW Australia; ^6^ Department of Renal Medicine Alfred Health Prahran Vic Australia; ^7^ Diabetes Australia Canberra ACT Australia; ^8^ Department of Diabetes and Endocrinology Royal North Shore Hospital St Leonards NSW Australia

**Keywords:** chronic kidney disease, diabetes, patient activation, self‐care, self‐management

## Abstract

**Objective:**

This study aimed to examine the association between performance of self‐care activities and patient or disease factors as well as patient activation levels in patients with diabetes and chronic kidney disease (CKD) in Australia.

**Methods:**

A cross‐sectional study was conducted among adults with diabetes and CKD (eGFR <60 mL/min/1.73m^2^) who were recruited from renal and diabetes clinics of four tertiary hospitals in Australia. Demographic and clinical data were collected, as well as responses to the Patient Activation Measure (PAM) and the Summary of Diabetes Self‐Care Activities (SDSCA) scale. Regression analyses were performed to determine the relationship between activation and performance of self‐care activities.

**Results:**

A total of 317 patients (70% men) with a mean age of 66.9 (SD=11.0) years participated. The mean (SD) PAM and composite SDSCA scores were 57.6 (15.5) % (range 0‐100) and 37.3 (11.2) (range 0‐70), respectively. Younger age, being male, advanced stages of CKD and shorter duration of diabetes were associated with lower scores in one or more self‐care components. Patient activation was positively associated with the composite SDSCA score, and in particular the domains of general diet and blood sugar checking (*P*<.05), but not specific diet, exercising and foot checking.

**Conclusion:**

In people with diabetes and CKD, a high level of patient activation was positively associated with a higher overall level of self‐care. Our results identify subgroups of people who may benefit from tailored interventions to further improve their health outcomes. Further prospective studies are warranted to confirm present findings.

## 1 INTRODUCTION

Patient activation specifies the level of patients’ involvement with their health care and refers to the extent to which they have the knowledge, motivation, belief, confidence and skills to manage chronic disease, access health care and to partner with health‐care providers for disease management.^1^, ^2^, ^3^ Patient activation is an important concept in chronic disease management driven by a person‐centred approach and chronic care models.^1^, ^4^ Higher levels of patient activation are associated with better patient outcomes compared to lower levels of activation, in chronic diseases.^1^, ^3^, ^5^, ^6^, ^7^ Individuals with low activation are more likely to be hospitalized,^8^, ^9^ have a longer length of stay in hospital,^10^ have greater health‐care costs,^11^ are less likely to participate in self‐management activities such as blood pressure monitoring^12^ and have worst care experiences^13^ compared to those with higher activation levels.

Patient self‐management is a patient's ability to participate in the management of symptoms, treatment and the physical, psychological and lifestyle consequences associated with chronic disease.^14^ There is growing evidence to suggest an association between patient activation levels and performance of self‐care activities for single chronic diseases including human immunodeficiency virus,^15^ congestive heart failure,^16^ schizophrenia^17^ and diabetes.^18^, ^19^ Patient activation predicts a variety of behaviours such as engaging in exercises, healthy diet and other disease‐specific self‐care and consumeristic behaviours.^6^, ^12^ However, studies are inconsistent in demonstrating an association between patient activation and self‐management for patients with diabetes and other long‐term diseases including chronic obstructive pulmonary disease (COPD), depression and musculoskeletal pain.^5^, ^12^, ^18^, ^20^

The PAM has previously been used as a screening tool for tailoring self‐management interventions or as a quality indicator for delivery of care.^21^ In the UK, one health service has redesigned the diabetes review process according to the individual's level of activation.^22^ Additionally, tailored coaching following activation assessment has resulted in improved clinical indicators and decreased health‐care utilization in patients with asthma, coronary artery disease, congestive heart failure, COPD and diabetes.^23^ Similarly, tailored care according to activation levels has been used to empower patients to ask questions during clinical reviews.^24^

There is a knowledge gap regarding the relationship between patient activation and self‐management in instances of comorbidity and multimorbidity such as diabetes and chronic kidney disease (CKD). This gap is important given that multimorbidity is increasing globally^25^, ^26^ and CKD commonly coexists with diabetes^27^ and is complex to manage. Moreover, greater understanding of how patient activation may influence performance of self‐care activities will be important in the design of interventions to increase self‐management.

The purpose of this study was to examine the association between performance of self‐care activities and patient or disease factors as well as patient activation levels in patients with diabetes and CKD.

## 2 METHODS

### 2.1 Study design and participants

The design and recruitment of participants for this study have been described in great detail previously.^28^ In short, patients attending diabetes and renal outpatient clinics of four public tertiary hospitals in the states of Victoria and New South Wales (Monash Health, Alfred Health, Royal North Shore Hospital and Concord Hospital) between 2013 and December 2014 were recruited.

Participants were included if they received their routine care at these hospitals and had a diagnosis of diabetes (either type 1 or type 2) and CKD stages 3‐5 (estimated glomerular filtration rate <60 mL/min/1.73 m^2^) including dialysis. Exclusion criteria included age less than 18 years of age, severe cognitive impairment and inability to communicate in English. Participants were identified as having diabetes if this was recorded from previous hospital records with the diagnosis of diabetes consistent with World Health Organization^29^ criteria.

Participants were recruited prospectively from clinics and completed the Patient Activation Measure (PAM‐13)^30^ and the Summary of Diabetes Self‐Care Activities (SDSCA)^31^ questionnaires (Supplementary Appendices A and B). Additionally, for each patient, a corresponding clinical survey was also completed by the site study staff or the clinician, using standardized procedures. Information obtained from the clinical survey included demographic characteristics such as age and gender. Disease‐specific characteristics such as diabetes duration, type of diabetes treatment, current HbA1c, CKD duration, CKD stage and current eGFR were also included (Supplementary Appendix C). The CKD EPI formula described by Levey and others^32^ was used to estimate eGFR. The units of measurement for eGFR were millilitre per minute per 1.73 m^2^.

Socio‐economic measures were estimated using the Australian Bureau of Statistics data.^33^ Postcodes were classified in accordance with the Index of Relative Social Disadvantage (IRSD), an index that provides a summary on a variety of data about the socio‐economic conditions of people living in an area.^33^ This was followed by categorizing the IRSD scores for each postcode into quintiles, where the lowest quintile represented 20% of postcodes with greatest socio‐economic deprivation. Written informed consent was obtained from all participants. The study received ethics approval from Monash University and the respective health service ethics committees.

### 2.2 Patient activation

The American version of the PAM‐13^30^ was used to evaluate the patients’ level of involvement in their health care. The PAM scale examines participants’ beliefs, knowledge and confidence in performing several self‐management activities and then yields a score based on patients’ answers to the 13 questions.^34^ There are four alternative responses to each of the 13 items namely, “disagree strongly, disagree, agree and agree strongly” and fifth response option “not applicable” (N/A) was available for all items.

The authors used a standardized spreadsheet provided by Insignia Health^®^ to calculate the PAM score.^35^ We excluded participants who responded to less than 7 items or if all questions were answered with “disagree strongly” or “agree strongly.” The mean PAM score was then calculated on all items leaving out the ones thought to be non‐applicable by the participants. The raw mean score was converted into a standardized activation score ranging from 0 to 100 creating the PAM scores which were classified into the four levels of activation: level 1 (score <47.0), level 2 (score 47.1‐55.1), level 3 (score 55.2‐67.0) and level 4 (score >67.0) as per Insignia Health^®^ scoring rules.^35^

### 2.3 Outcomes

Self‐management was evaluated by the SDSCA questionnaire,^31^ a self‐report measure of how often participants perform diabetes self‐care activities. The SDSCA questionnaire has been utilized in several studies and settings and is deemed to be reliable, valid and sensitive^36^, ^37^, ^38^ in evaluating diabetes self‐management in adults. This study used a version of the SDSCA questionnaire that comprised of items assessing five domains of diabetes self‐management which are “general diet (2 items), specific diet (2 items), exercise (2 items), blood glucose testing (2 items) and foot care (2 items)”.^31^ The medication self‐management component was excluded based on previous reports of its “ceiling effects and lack of variability among participants”.^31^ The smoking self‐management component was also excluded because smoking behaviour was relevant to smokers only.

### 2.4 Statistical analysis

Results are presented as mean and standard deviation (SD) and median and interquartile range (IQR) for normal and non‐normally distributed data, respectively. Duration of diabetes was categorized into quartiles. First, chi‐squared or t tests (as appropriate) examined differences in patient and disease characteristics by performance of self‐care activities and levels of patient activation using PAM score as a continuous variable. Second, chi‐squared tests for linear trend examined differences in performance of self‐care activities across the four levels of patient activation (PAM score categories 1‐4). Third, univariable and multivariable linear regression models assessed the relationship between the performance of self‐care activities (composite SDSCA score) and the four levels of patient activation (PAM score categories 1‐4), and any potential effect of patient or disease characteristics (any variable with a *P* value of <.1 in the univariable analysis). Similar models assessed the relationship between the individual self‐care activities and the four levels of patient activation. A sensitivity analysis examined the effect of substitution of PAM score as a continuous variable into the models. All analyses were performed with Stata version 11 (Statacorp, College Station, TX). Statistical significance was indicated by a *P* value of <.05.

## 3 RESULTS

### 3.1 Patient characteristics

**Figure 1:**
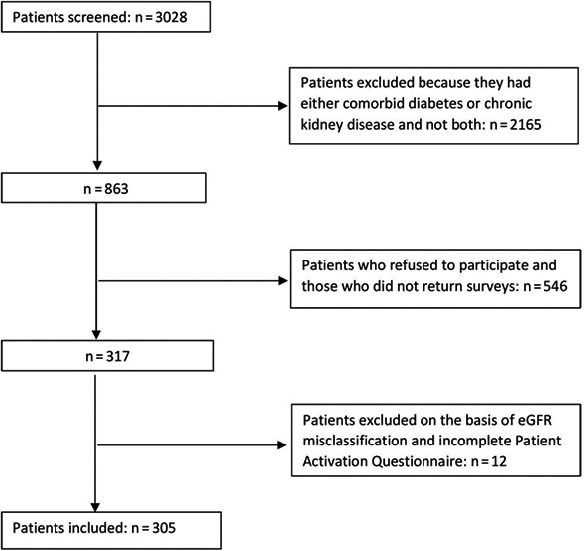
Patient inclusion flow diagram

A total of 3028 patients were screened and 305 were included in the analyses after exclusion of nine patients who had their eGFR misclassified (>60 mL/min/m^2^) and three patients who had incomplete PAM data (Figure 1). There were no differences in age, gender and stage of kidney disease between responders and non‐responders (Table S1). Participants’ age ranged from 32 to 90 years (median 68 years), with a predominance of men (70% of all participants). The mean (SD) PAM and composite SDSCA scores were 57.6 (15.5) % (range 0‐100) and 37.3 (11.2) (range 0‐70), respectively (Table 1). Approximately 50% of participants were of upper socio‐economic status. Patient activation did not significantly differ by gender, age, socio‐economic status, CKD stage, dialysis status, diabetes, and CKD duration (Table 1). Participation in self‐care activities did not significantly differ by any demographic and clinical characteristics except for diabetes duration (*P*<.05).

**Table 1:** Demographic and clinical characteristics for all patients by mean activation and composite SDSCA scores

	Total N (%)	Mean PAM scores (SD) Range (0‐100)	Mean composite SDSCA scores (SD) Range (0‐70)
Total	305 (100)	57.6 (15.5)	37.3 (11.2)
Gender			
Male^a^	212 (69.5)	57.4 (15.9)	36.7 (11.5)
Female^b^	93 (30.5)	58.1 (14.4)	38.2 (10.3)
Age			
<68 y	156 (51.1)	57.2 (15.0)	37.2 (11.3)
>68 y	149 (48.9)	58.0 (16.0)	37.2 (11.3)
Socio‐economic status			
Upper	160 (53.2)	58.0 (16.3)	37.2 (11.4)
Upper middle	40 (13.3)	54.8 (17.2)	37.3 (9.8)
Lower middle	49 (16.3)	58.0 (13.7)	36.0 (11.1)
Upper lower	21 (7.0)	58.0 (15.6)	36.0 (11.1)
Lower	31 (10.3)	56.3 (10.2)	38.5 (10.3)
Smoking status			
Yes	18 (5.9)	58.5 (11.3)	34.8 (12.7)
No	287 (94.1)	57.5 (15.7)	37.3 (12.7)
Stage of CKD^c^			
3a	72 (23.6)	59.2 (15.9)	37.4 (11.3)
3b	79 (25.9)	58.6 (17.8)	39.5 (10.5)
4	74 (24.3)	57.5 (15.1)	35.0 (11.0)
5	80 (26.2)	55.4 (12.7)	36.9 (11.3)
Dialysis			
Yes	59 (19.3)	55.5 (13.0)	38.4 (9.6)
No	246 (80.7)	58.1 (16.0)	36.9 (11.5)
Diabetes duration			
0‐8 y	81 (26.6)	58.6 (16.1)	34.5 (12.5)*
9‐18 y	80 (26.2)	54.6 (15.2)	36.6 (11.9)
19‐25 y	80 (26.2)	59.8 (13.7)	38.4 (9.5)
26 y and over	64 (21.0)	57.3 (16.9)	40.0 (9.3)
Kidney disease duration			
<5 y	125 (41.0)	58.1 (15.3)	37.9 (11.6)
>5 y	180 (59.0)	57.3 (15.6)	36.7 (10.7)

^a^Missing PAM data for two male participants, not included in analysis; ^b^Missing PAM data for one female participant, not included in analysis; ^c^Kidney Disease Outcomes Quality Initiative staging of CKD based on GFR, 3a (45‐59), 3b (30‐44), 4 (15‐29) 5 (less than 15 or on dialysis); age and CKD duration were stratified by median and diabetes duration by quartiles; *P<.05.

### 3.2 Association between self‐care activities and patient or disease factors

**Figure 2:**
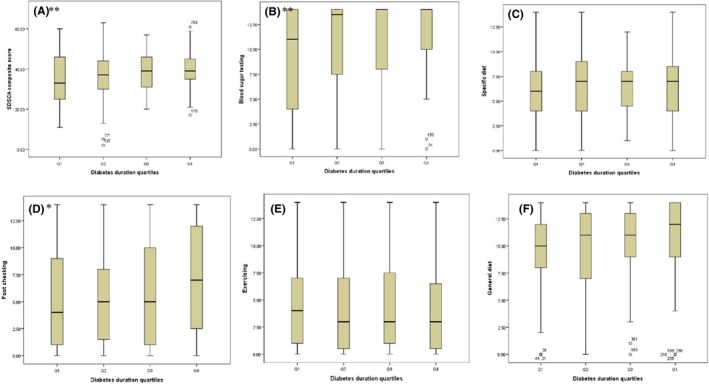
A‐F, Nonparametric test for trend assessing differences in self‐management practices across diabetes duration quartiles. **P<.01 and *P<.05

Patient factors associated with self‐care activities are shown in Table 2. On multivariable analysis, younger age was associated with lower scores in the general diet domain (all *P* value <.05). Male patients had lower scores in the blood sugar checking domain where they scored 1.6 points less than female patients. A shorter duration of diabetes was associated with lower composite scores, and with lower scores in the blood sugar checking and foot checking domains (all *P*<.05). (Figure 2). Patients with stage 5 kidney disease scored 1 point less than patients with stage 3a disease in the exercising domains. No association was found between socio‐economic status and the composite score or any specific self‐care domain.

**Table 2:** Summary of factors predicting self‐management behaviours in patients with diabetes and chronic kidney disease

Covariates	Composite self‐management score		General diet		Specific diet	
	Univariable	Multivariable	Univariable	Multivariable	Univariable	Multivariable
	B (95% CI)		B (95% CI)		B (95% CI)	
Age	0.05 (−0.02; 0.09)	‐	0.1 (0.02; 0.1)**	0.06 (0.02; 0.09)**	−0.003 (−0.03; 0.03)	‐
Gender						
Male	1 (ref)		1 (ref)		1 (ref)	
Female	1.4 (−1.3; 4.2)	‐	0.4 (−0.5; 1.3)	‐	−0.1 (−0.8; 0.7)	‐
SES (quintiles)						
Upper	1 (ref)		1 (ref)		1 (ref)	
Upper middle	−1.4 (−5.4; −2.6)	‐	−1.1 (−2.4; 0.2)	‐	0.5 (−1.6; 0.6)	‐
Lower middle	−0.04 (−4.5; −3.7)	‐	−0.04 (−1.4; 1.3)	‐	0.2 (−0.9; 1.3)	‐
Upper lower	−1.7 (−5.7; −2.4)	‐	−0.4 (−1.7; 1.0)	‐	−0.4 (−1.5; 0.7)	‐
Lower	−0.6 (−4.6; −3.5)	‐	−0.6 (−2.0; 0.7)	‐	0.1 (−1.0; 1.2)	‐
DM duration	0.2 (0.1; 0.3)**	0.2 (0.1; 0.3)**	0.02 (−0.02;0.1)	‐	0.01 (−0.02; 0.04)	‐
CKD duration	−0.03 (−0.2; 0.1)	‐	−0.001 (−0.05; 0.04)		−0.03 (−0.06; 0.01)	‐
Stage of CKD						
3a	1 (ref)	‐	1 (ref)		1 (ref)	
3b	2.5 (−1.1; 6.1)	‐	0.8 (−0.4; 2.0)	‐	0.5 (−0.5; 1.5)	‐
4	−2.2 (−5.9; 1.4)	‐	0.6 (−0.7; 1.8)	‐	−0.1 (−1.0; 0.9)	‐
5	−0.5 (−4.1; 3.1)	‐	0.2 (−1.0; 1.4)	‐	0.9 (−0.1; 1.8)	‐
PAM levels						
4	1 (ref)		1 (ref)		1 (ref)	
3	−3.6 (−7.2; −0.1)*	−4.1 (−7.6; −0.6)*	−1.7 (−3.1; −0.4)*	−1.1 (−2.3; 0.1)	0.4 (−0.6; 1.3)	‐
2	−4.9 (−8.7; −0.1)*	−5.3 (−9.1; −1.8)**	−1.3 (−2.6; 0.003)*	−1.3 (−2.6; −0.01)*	0.4 (−0.7; 1.4)	‐
1	−5.8 (−9.7; −1.9)**	−5.6 (−9.5; −1.8)**	−1.2 (−2.4; 0.04)	−1.8 (−3.1; −0.5)**	−0.2 (−1.2; 0.9)	‐

Covariates	Exercising		Blood sugar checking		Foot checking	
	Univariable	Multivariable	Univariable	Multivariable	Univariable	Multivariable
	B (95% CI)		B (95% CI)		B (95% CI)	
Age	−0.01 (−0.05; 0.03)	‐	0.01 (−0.04; 0.1)	−	0.01 (−0.04; 0.1)	‐
Gender						
Male	1 (ref)		1 (ref)		1 (ref)	
Female	−0.6 (−1.6; 0.3)	‐	1.4 (0.3; 2.5)*	1.6 (0.5; 2.7)*	0.7 (−0.5; 1.9)	‐
SES (quintiles)						
Upper	1 (ref)		1 (ref)		1 (ref)	
Upper middle	0.2 (−1.2; 1.6)	‐	−0.2 (−1.8; 1.4)	‐	0.4 (−1.3; 2.1)	‐
Lower middle	0.2 (−1.2; 1.7)	‐	−0.3 (−2.0; 1.3)	‐	−1.4 (−3.2; 0.3)	‐
Upper lower	−0.1 (−1.5; 1.3)	‐	−0.1 (−1.8; 1.6)	‐	−1.0 (−2.7; 0.7)	‐
Lower	0.3 (−1.1; 1.7)	‐	−0.3 (−2.0; 1.3)	‐	0.02 (−1.7; 1.7)	‐
DM duration	−0.01 (−0.05; 0.03)	‐	0.1 (0.06; 0.2)***	0.1 (0.07; 0.2)***	0.1 (0.01; 0.1)**	0.1 (0.01; 0.1)*
CKD duration	−0.02 (−0.1; 0.03)	‐	−0.01 (−0.1; 0.1)	‐	0.02 (−0.04; 0.1)	‐
Stage of CKD						
3a	1 (ref)	‐	1 (ref)		1 (ref)	
3b	−0.1 (−1.4; 1.1)	‐	0.7 (−0.7; 2.2)	‐	0.1 (−1.5; 1.5)	‐
4	−0.8 (−2.1; 0.4)	‐	−0.6 (−2.1; 0.9)	‐	−1.8 (−3.3; −0.2)**	−1.7 (−3.0; −0.5)**
5	−1.6 (−2.9; −0.4)*	−1.3 (−2.3; −0.3)*	−0.3 (−1.8; 1.1)	‐	−0.1 (−1.6; 1.4)	‐
PAM levels						
4	1 (ref)		1 (ref)		1 (ref)	
3	−1.0 (−2.3; 0.2)	‐	−1.1 (−2.6; 0.3)	−1.5 (−2.9; −0.1)*	−0.8 (−2.3; 0.8)	‐
2	−1.5 (−2.8; −0.1)*	‐	−1.9 (−3.5; −0.3)*	−2.1 (−3.7; −0.6)**	−0.9 (−2.6; 0.7)	‐
1	−1.3 (−2.6; 0.1)	‐	−2.0 (−3.7; −0.4)*	−1.9 (−3.5; −0.4)*	−0.9 (−2.6; 0.8)	‐

*P<.05; **P<.01; ***P<.001; SES—socio‐economic status; DM—diabetes mellitus; CVD cardiovascular disease CKD—chronic kidney disease; PAM—patient activation measure; B (95% CI)—confidence intervals for beta coefficients, which represent the amount that the dependent variable (SDSCA domains) changes when the independent variable changes by 1 unit.

### 3.3 Association between self‐care activities and patient activation

**Figure 3:**
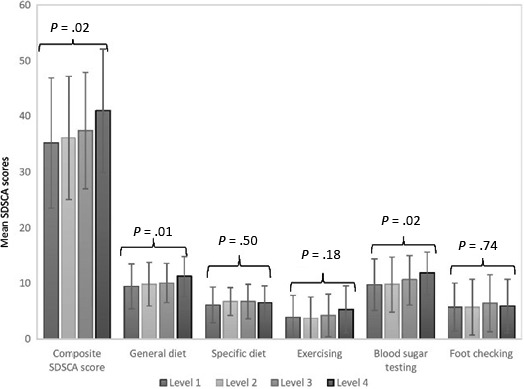
Mean scores of the composite SDSCA scale and the five individual SDSCA domains across ordered levels of patient activation. Statistical significance values are for trend across patient activation levels 1 to 4

With decreasing patient activation level, the mean scores for the composite self‐care score and the domains of general diet and blood sugar testing (all *P*<.05) decreased significantly, whereas the mean scores for the domains of specific diet, exercising and foot checking did not (Figure 3). Patients with level 1 activation scored 2‐6 points lower than patients with level 4 activation (reference group) for the composite score, and the domains of general diet and blood sugar testing (Table 2).

In univariable and multivariable analyses, the level of patient activation was positively associated with the composite self‐care score and the domains of general diet and blood sugar checking (all *P*<.05) but not the domains of specific diet, exercising and foot checking (Table 2). When patient activation was included in the models as a continuous variable, the results remained similar (data not shown).

## 4 DISCUSSION

In our study, among patients with comorbid diabetes and CKD, we have demonstrated an association between patient activation and diabetes self‐care activities. A higher patient activation level was associated with a higher overall self‐care score. However, this association was not observed for all specific self‐care domains; only for general diet and blood sugar checking. Additionally, different patient and disease characteristics were associated with diabetes self‐care: younger age and male gender were associated with less home blood glucose monitoring, more severe CKD was associated with less foot checking and exercising, and a shorter duration of diabetes was associated with lower overall self‐care score as well as less blood sugar checking and foot checking.

In patients with comorbid diabetes and CKD, higher patient activation levels were associated with higher composite self‐care scores. Previous studies have only examined this association for single chronic diseases, such as diabetes.^18^ In patients with diabetes, the relationship is inconsistent, with some studies showing a positive association between patient activation and self‐care activities^5^, ^39^ and others showing no association.^18^ In patients with CKD, this association has not been explored. Our study adds to the literature by showing that in the setting of multimorbidity, the association is positive and independent of certain potential confounding patient or disease factors such as age, gender and disease duration.

Interestingly, the association between patient activation levels and diabetes self‐care was not observed for all specific self‐care domains. While there was a positive association between general diet and blood sugar checking, there was no association between patient activation levels and specific diet, exercising and foot checking domains. This suggests that an activated patient may not necessarily or automatically participate in all self‐care activities—they not only need to have knowledge, motivation and skills to self‐manage, but they also need to have the physical and financial ability to self‐manage across all domains of diabetes self‐management. A possible reason for the lack of association between PAM and exercise and foot checking is that both these activities require a certain degree of physical fitness and ability, which is compromised in patients with diabetes and CKD due to comorbidity.^40^, ^41^ Similarly, a lack of association between PAM and a specific diet could be that the specific diabetes diet may be financially prohibitive.^42^, ^43^ These results highlight the importance of addressing all self‐care domains to improve self‐management for patients with comorbid diabetes and CKD across all spectrums of activation.

We found an association between younger ages and lower self‐care scores in the domain of general diet independent of patient activation. The explanation is likely to be multifactorial, but we hypothesize that younger patients may be less motivated to self‐manage compared to older patients, as risk perception is altered in younger populations, especially in males^44^, ^45^ and they have competitive priorities that take precedence such as socializing and work commitments.^46^ Lack of knowledge may also contribute but less so than other factors given that younger patients are reported to have greater diabetes knowledge than older patients.^47^

Additionally, we found that a shorter duration of diabetes was associated with lower self‐care scores. Previous studies among patients with diabetes have not been consistent with some reporting an association between lower self‐care scores with a shorter duration of diabetes,^48^, ^49^ while others reported an association between lower self‐care scores and longer duration of diabetes.^50^, ^51^ In patients with comorbid diabetes and CKD, we found a shorter duration of diabetes to be associated with lower self‐care scores. This suggests that patients with a shorter duration of diabetes may not be exposed to sufficient diabetes education or have not yet mastered self‐management skills, and should be targeted by interventions to improve self‐management such as tailored Diabetes Self‐Management Education and support^52^. Alternatively, participants with a longer duration of diabetes are likely to be older and may have some physical limitations such that they receive more attention and social support to improve their ability to self‐manage.^53^

More advanced CKD was associated with lower scores in the self‐care domains of exercising and foot checking. Exercising and foot checking require a certain level of mobility and physical fitness such that patients with advanced CKD with lower exercise tolerance, and functional capacity, and more muscle wasting cannot as easily complete self‐care activities without assistance.^54^, ^55^ This emphasizes the importance of the actual physical fitness of an individual in performing self‐care activities and is an important factor to consider when individualizing management of a patient with advanced diabetes and CKD.

Our findings should be interpreted in the light of the strengths and limitations of our study. The strengths include that our study provides insight into the level of activation and utility of the PAM in patients with diabetes and moderate to severe CKD, a group of patients who may have a greater need for support to engage in their health‐care needs. Our data are consistent with and extends the findings of previous longitudinal studies by assessing patients across different stages of CKD. This informs the provision of targeted interventions to improve the activation levels of patients with more advanced renal disease. The other strengths include the inclusion of several demographic and clinical variables as potential predictors for diabetes self‐management behaviour, and the use of valid and reliable tools to measure patient activation^30^ and diabetes self‐management.^31^ Additionally, the study population was drawn from multiple hospitals across Australia, increasing generalizability of our findings. Potential limitations are due to the cross‐sectional nature of the study design, which did not allow us to track patient activation patterns over time. Assessment of patient activation over time permits an early identification of patients in whom a change in activation levels may flag a change in health status. Moreover, longitudinal PAM data can be used to develop risk prediction models that predict adverse patient outcomes.^56^ Another apparent limitation was the modest response rate of 38.5%, which is, however, comparable to other studies in people with diabetes.^18^, ^57^ We did not collect data on some factors such as depression and health literacy, which have been found to be associated with patient activation in different population groups.^58^, ^59^ In addition, our sample of participants who attend hospital may be a biased group from the aspect of utilizers of the service.

Our findings have important implications for practice and future research. First, targeted multifactorial risk reduction interventions focusing on subgroups of patients identified in this study, who are likely to perform poorly in self‐care activities, may improve health outcomes. There is evidence that such interventions could be delivered optimally through collaborative care,^60^ a key feature of combined diabetes kidney specialist clinics, which often have a multidisciplinary team.^61^ Second, we have shown that highly activated patients are more likely to participate in self‐care activities than those with low activation levels. Additionally, assessment of patient activation in this patient group, which is already suffering a double burden of chronic disease,^62^, ^63^ ensures that resources are directed to those who need them most, thereby improving on resource utilization and reduction in health inequalities. Our study, being of an exploratory nature, opens up opportunities for future research, which should include well‐designed and disease‐specific longitudinal studies to validate and extend our findings.

In patients with comorbid diabetes and CKD, although a high level of patient activation in self‐care is associated with a high level of patient self‐management in general, this is not the case across all individual domains of diabetes self‐care. Patient age, gender, duration of diabetes and stage of CKD may also influence patient self‐management in comorbid diabetes and CKD.

## CONFLICTS OF INTEREST

The authors declare no conflicts of interest in relation to this work.
